# Maternal death and delays in accessing emergency obstetric care in Mozambique

**DOI:** 10.1186/s12884-018-1699-z

**Published:** 2018-03-22

**Authors:** Leonardo Antonio Chavane, Patricia Bailey, Osvaldo Loquiha, Martinho Dgedge, Marc Aerts, Marleen Temmerman

**Affiliations:** 1 Jhpiego Mozambique, Rua A.W. Bayly, 61 Maputo, Mozambique; 2 RMNCH Unit, Global Health Programs, FHI, Durham, NC 360 USA; 3 Department of Mathematics and Informatics, Eduardo Mondlane University, Maputo, Mozambique; 4 Faculty of Medicine, Eduardo Mondlane University, Maputo, Mozambique; 5 Interuniversity Institute for Biostatistics and Statistical Bioinformatics (I–BioStat), Hasselt University, Hasselt, Belgium; 6 Centre of Excellence in Women and Child Health- East Africa, Aga Khan University, Karachi, Pakistan; 7 Ghent University Belgium, Ghent, Belgium

**Keywords:** Maternal deaths, Delays type II & III, Mozambique

## Abstract

**Background:**

Despite declining trends maternal mortality remains an important public health issue in Mozambique. The delays to reach an appropriate health facility and receive care faced by woman with pregnancy related complications play an important role in the occurrence of these deaths. This study aims to examine the contribution of the delays in relation to the causes of maternal death in facilities in Mozambique.

**Methods:**

Secondary analysis was performed on data from a national assessment on maternal and neonatal health that included in-depth maternal death reviews, using patient files and facility records with the most comprehensive information available. Statistical models were used to assess the association between delay to reach the health facility that provides emergency obstetric care (delay type II) and delay in receiving appropriate care once reaching the health facility providing emergency obstetric care (delay type III) and the cause of maternal death within the health facility.

**Results:**

Data were available for 712 of 2,198 maternal deaths. Delay type II was observed in 40.4% of maternal deaths and delay type III in 14.2%.and 13.9% had both delays. Women who died of a direct obstetric complication were more likely to have experienced a delay type III than women who died due to indirect causes. Women who experienced delay type II were less likely to have also delay type III and vice versa.

**Conclusions:**

The delays in reaching and receiving appropriate facility-based care for women facing pregnancy related complications in Mozambique contribute significantly to maternal mortality. Securing referral linkages and health facility readiness for rapid and correct patient management are needed to reduce the impact of these delays within the health system.

## Background

### Maternal death and delays in accessing emergency obstetric care in Mozambique

Delays in access to quality care have been identified as one of the important determinants of preventable maternal death [1, 2]. Thaddeus and Maine’s three delays model that describes the multiple factors that drive maternal mortality has proven to be an effective tool to evaluate the circumstances surrounding access to and appropriateness of emergency obstetric and newborn care (EmONC) [3]. The model has been helpful identifying barriers and potential points of intervention along the continuum from home to hospital for more than 20 years [4–6].

According to this framework 3 delays in access to quality emergency care are defined. The first delay occurs at the household and community level and reflects the delay in deciding to seek care for pregnancy complications. The second delay (delay II) refers to the delay to reach the facility that provides emergency obstetric care (EmONC) and, the third delay (delay III) refers to the delay that occurs in receiving care after arrival at the health facility [3].

In Mozambique, estimates based on the 2007 population census indicate that around 46% of maternal deaths occur within health institutions [7], a substantial proportion considering that these women reached a health facility.

Maternal mortality ratio in Mozambique has declined from more than 1600 estimated deaths per 100.000 live births in 1990 [8] to 403 in 2003 [9] and DHS 2011 [10]. Despite considerable reduction over time, the latest estimations available show that the ratio still is very high, even when compared with the neighboring countries [2].

Several factors have been identified at the health care facility level that interfere with the readiness to deal adequately with obstetric emergencies. Knight described six groups of factors, namely drugs and equipment, policy and guidelines, human resources, facility infrastructure, patient-related and referral-related aspects [11].

A near miss study, an approach to evaluate maternal quality of care and learning from women that survived severe maternal complications [12, 13] covered 564 survival women and 71 maternal deaths, conducted in Mozambique’s Maputo Province, found delay II in 21.3% and delay III in 69.7% (women could experience multiple delays [14]. In some cases these delays followed the woman’s path throughout the referral system from admission to a peripheral health facility and then on to the referral facility [14].

With this paper we aim to examine the relationship between the cause of maternal death and delays II and III.

## Methods

### Study setting

Mozambique is a low income country located in the Sub-Saharan Africa region with an estimated population of 26.4 million in 2016, based on projections of the 2007 population census. The public sector is the main provider of health care services [15]. Childbirth services are provided throughout 4 levels of care. At peripheral level services are available in the smallest type 2 health centers, with 1 MCH nurse as provider and 3 maternity beds. The different layers of care are connected by a network referral system based on ambulances managed at the referral health facilities. Usually a health facility covering an average population of 300.000 inhabitants has 1 to 2 ambulances to serve a network of 10 to 15 health facilities. In the district capitals maternity services are available in large type 1 health centers, with more than 10 maternity beds; at this level the staff include at least 3 MCH nurses and in some facilities, a general practitioner physician. The District, Provincial, General and Central hospitals are more complex facilities with different specialties, wards and operating rooms. Health centers type 1 and 2 should have capacity to offer Basic Emergency Obstetric Care (BEmOC). According to the Annual Joint Assessment Report in 2012, in average 2.3 health facilities were available with BEmOC services per 500 thousand inhabitants. When needed, the health centers refer patients to hospitals with capacity to offer Comprehensive Emergency Obstetric Care (CEmOC).

This paper draws on secondary data analyses of a large health facility needs assessment on maternal and neonatal health care services, which included 427 health facilities nationwide. The sample was designed to be representative of the mix of the health facilities at national and provincial levels. Data collection was done between November 2007 and January 2008 by health providers previously trained to undertake this task. Service statistics were collected from registers and logbooks and covered a period of 12 months, from November 2006 to October 2007.

The national survey identified a total of 2,198 maternal deaths in the 427 health facilities out of 312,537 deliveries, corresponding to an estimated facility based maternal mortality ratio of 703 deaths per 100.000 deliveries. At each facility, a detailed maternal death review was conducted if the patient’s records were available. A maternal death review was completed for 712 maternal deaths for which there was sufficient information in the files. Prior to data entry, these were reviewed by a committee of experts composed of 6 ObGyn Medical doctors. The committee reviewed the circumstances of death to determine: 1) the final cause of death, 2) if evidence existed to suggest that the woman experienced a delay in arriving at the health facility providing emergency obstetric care services (delay II). Women that faced delay in the referral process reaching the second health facility was considered as delay II. and 3) if evidence suggested a delay in receiving adequate health care services after reaching the health facility with emergency obstetric care services (delay III).

For each maternal death, the time of arrival at the initial health facility was collected as well as the time of treatment initiation, time of arrival at the referral (second) facility and time of death. The delay was defined based on assessment of each woman’s individual story since her arrival at the initial health facility. This included the combination of the clinical status on admission in the health facility, and the time taken for action at peripheral and referral facilities. Women presenting life threatening condition at admission of ether initial facility or at the referral facility were considered facing delay type II. In all these steps the quality of care was assessed relating the decision (diagnosis and or to refer to next level of care) and the treatment prescribed and or initiated. The 712 maternal deaths were found in 93 of the 427 facilities included in the survey, including health centers and hospitals, located in rural and urban areas of the 11 provinces of Mozambique. Approval from the Mozambican national bioethical committee was received before starting the survey implementation.

### Statistical analysis

Data were analyzed using the SAS-STAT software, version 9.4. A bivariate binary mixed effects model was applied to examine if and how the probability for delay type II and delay type III depends on the main cause of maternal death and other risk factors and covariates, while accounting for the correlation between both types of delay. Specifically, as in Wilson and Lorenz (2015) [16],we used one logistic regression model for each outcome variable (delay type II and III), both models consisting of fixed effects related to observed covariates and a random intercept related to unobserved effects affecting both delay types specific to each woman.

The advantage of such model specification is that it allows the logistic regressions to be modelled simultaneously, for which, conditional on the latent variable, delay type II and III were assumed as independent outcomes. This allows the estimation of the correlation between the outcomes while also controlling for the main cause of death and other observed covariates such as age, parity, referrals, type of health facility and availability of human resources. The potential covariates were selected for the final model using logistic regression for each outcome separately in a backwards selection procedure with removal rob of 0.2. The initial model didn’t include any interaction between the risk factors.

Inference was based on 95% confidence intervals and *p*-values for odds ratio estimates, and these were only valid if the missing data mechanism was known to be missing completely at random**, (**MCAR) [17, 18]. About 13% of cases with missing data for either outcome were excluded from the analysis (see Table 1 for more details), and considered to be MCAR, since being missing was not related to any covariate investigated in this study. The latter was based on a logistic regression model on the missing indicator for both delay types (results not shown), for which no significant association was found between the missing indicator and the covariates [17].

**Table 1:** Summary of selected characteristics of maternal deaths

		Neither delay II nor delay III		Delay III only		Delay II only		Both delay II and delay III		Missing		Total	
		Count	Row N %	Count	Row N %	Count	Row N %	Count	Row N %	Count	Row N %	Count	Column N %
Age recoded	< 20	25	18.7%	22	16.4%	46	34.3%	21	15.7%	20	14.9%	134	18.8%
	20 -- 34	90	20.4%	54	12.2%	190	43.0%	56	12.7%	52	11.8%	442	62.1%
	> 35	14	15.9%	17	19.3%	37	42.0%	13	14.8%	7	8.0%	88	12.4%
	Missing	3	6.3%	8	16.7%	15	31.3%	9	18.8%	13	27.1%	48	6.7%
Parity	Nullipara	29	22.1%	20	15.3%	37	28.2%	18	13.7%	27	20.6%	131	18.4%
	> 1	96	19.1%	76	15.1%	205	40.8%	68	13.5%	57	11.4%	502	70.5%
	Missing	7	8.9%	5	6.3%	46	58.2%	13	16.5%	8	10.1%	79	11.1%
Previous cesarean	No	101	19.3%	77	14.8%	207	39.7%	69	13.2%	68	13.0%	522	73.3%
	Yes	11	22.0%	8	16.0%	21	42.0%	5	10.0%	5	10.0%	50	7.0%
	Missing	20	14.3%	16	11.4%	60	42.9%	25	17.9%	19	13.6%	140	19.7%
Complication after admission	No	88	15.8%	67	12.0%	262	47.0%	88	15.8%	53	9.5%	558	78.4%
	Yes	29	39.2%	21	28.4%	6	8.1%	2	2.7%	16	21.6%	74	10.4%
	Missing	15	18.8%	13	16.3%	20	25.0%	9	11.3%	23	28.8%	80	11.2%
Referred from another facility	No	62	19.7%	61	19.4%	113	35.9%	30	9.5%	49	15.6%	315	44.2%
	Yes	67	17.7%	39	10.3%	171	45.2%	67	17.7%	34	9.0%	378	53.1%
	Missing	3	15.8%	1	5.3%	4	21.1%	2	10.5%	9	47.4%	19	2.7%
Type of Health facility	Central Hospital	40	19.7%	13	6.4%	96	47.3%	39	19.2%	15	7.4%	203	28.5%
	Hospital (Prov, Gen. & Dist.)	64	16.6%	64	16.6%	153	39.6%	48	12.4%	57	14.8%	386	54.2%
	Health Centers	28	22.8%	24	19.5%	39	31.7%	12	9.8%	20	16.3%	123	17.3%
Moment of death	While pregnant	31	14.2%	28	12.8%	101	46.1%	32	14.6%	27	12.3%	219	30.8%
	During delivery	10	10.3%	21	21.6%	26	26.8%	18	18.6%	22	22.7%	97	13.6%
	Postpartum	88	23.5%	50	13.3%	154	41.1%	48	12.8%	35	9.3%	375	52.7%
	Missing	3	14.3%	2	9.5%	7	33.3%	1	4.8%	8	38.1%	21	2.9%
*Cause of death	Direct	98	18.7%	85	16.2%	200	38.1%	86	16.4%	56	10.7%	525	73.7%
	Indirect	34	18.2%	16	8.6%	88	47.1%	13	7.0%	36	19.3%	187	26.3%
	Total	132	18.5%	101	14.2%	288	40.4%	99	13.9%	92	12.9%	712	100.0%

*Cause of death: Direct = Maternal death from direct pregnancy related complications; Indirect = Maternal deaths due to non-obstetric causes aggravated by the pregnancy

*Prov* Province, *Gen* general, *Dist* district

## Results

Table 1 below presents the women’s demographic characteristics, previous obstetric history, facility characteristics, timing and cause of death (direct or indirect). Also this table presents the distribution of proportion of delays within the selected characteristics.

Most women (62.1%) were between 20 and 34 years old at the time of their death. The median age was 25 years and only 18.4% were nulliparous. Most women arrived at the health facility with an obstetric complication (78.4%), and 53.1% were referred from another health facility. Delay II (reaching the health facility where she died) was observed in 40.4% of deaths and delay III (receiving treatment after arrival at the health facility) was registered in 14.2% of cases, 13.9 faced the both type of delays and in 18.5% of cases no evidence of delay was identified. Direct obstetric complications were registered as the main cause of death for most cases (73.7%).

Around 10% of women developed complications after admission in the health facilities. Out of this group, 53% of deaths occurred at the tertiary hospitals while 35% at the health Centers and 12% at the Central Hospitals. The main causes of death in this group were post-partum hemorrhage which accounted for 28% followed by uterine rupture responsible of 14% of deaths. Uterine rupture was the first cause at the health centers level.

Table 2 summarizes the causes of death in relation to the type of delay. Amongst the direct causes of death, hemorrhage was observed in 60.6% of cases, followed by sepsis in 20.8%, while HIV/AIDS was frequent amongst the indirect causes, observed in 40.6% of the cases**.**

**Table 2:** Delays type II and type III by cause of death

	Neither delay II nor III		Delay III only		Delay II only		Both delay II and III		Missing		Total	
	n	%	n	%	n	%	n	%	n	%	n	%
Direct causes	98	18.7	85	16.2	200	38.1	86	16.4	56	10.7	525	73.7
Hypertension	21	23.3	12	13.3	40	44.4	10	11.1	7	7.8	90	17.1
Hemorrhage	59	18.6	53	16.7	116	36.5	50	15.7	40	12.6	318	60.6
Sepsis/Infections	14	12.8	18	16.5	44	40.4	25	22.9	8	7.3	109	20.8
Other direct causes	4	50	2	25	0	0	1	12.5	1	12.5	8	1.5
Indirect causes	34	18.2	16	8.6	88	47.1	13	7	36	19.3	187	26.3
Malaria	8	15.4	7	13.5	22	42.3	7	13.5	8	15.4	52	27.8
HIV/AIDS	14	18.4	2	2.6	50	65.8	0	0	10	13.2	76	40.6
Malaria/HIV-AIDS	2	16.7	0	0	7	58.3	1	8.3	2	16.7	12	6.4
Severe anemia	1	7.7	2	15.4	4	30.8	3	23.1	3	23.1	13	6.9
Other indirect causes	6	26.1	3	13	5	21.7	1	4.4	8	34.8	23	12.3
Unknown/Undetermined	3	27.3	2	18.2	0	0	1	9.1	5	45.5	11	5.9
Total	132	18.5	101	14.2	288	40.4	99	13.9	92	12.9	712	100%

Around 40.4% of women suffered delay type II but not delay type III, 13.9% experienced both delay type II and type III and 18.5% did not experienced any type of delay at the referral facility.

The modelling results in Table 3 show a significant association between the main cause of death and delay type III after controlling for other risk factors. Pregnant women who died of a direct pregnancy complication were twice more likely to have experienced a delay type III than pregnant women who died due to indirect causes. However, no significant association between delay type II and cause of death was observed.

**Table 3:** Parameter estimates for the bivariate logistic regression model (with random intercepts)

	Delay II		Delay III		Overall effect
Effect	O.R. (95% C.I)	p-value	O.R. (95% C.I)	p-value	p-value
Age					
< 20	Ref.		Ref.		
20 -- 34	1.32 (0.82 -- 2.12)	0.2565	**0.59** (0.37 -- 0.96)	**0.0324**	**0.0345**
> 35	1.21 (0.62 -- 2.37)	0.5729	0.73 (0.38 -- 1.42)	0.3545	0.3586
Parity					
Nullipara	Ref.		Ref.		
> 1	0.80 (0.51 -- 1.26)	0.3343	1.17 (0.74 -- 1.84)	0.5109	0.297
Referred					
No	Ref.		Ref.		
Yes	**1.76** (1.18 -- 2.62)	**0.0055**	0.67 (0.45 -- 1.00)	0.0506	**0.0019**
Type of Health Facility					
Health Centers (I;II)	Ref.		Ref.		
Central Hospital	**2.37** (1.30 -- 4.33)	**0.0049**	0.89 (0.49 -- 1.62)	0.6957	**0.0279**
Prov, Gen & Dist Hosp.	1.52 (0.90 -- 2.58)	0.1207	1.16 (0.68 -- 1.99)	0.5839	0.4603
Cause of death					
Indirect	Ref.		Ref.		
Direct	0.66 (0.42 -- 1.05)	0.0771	**2.11** (1.29 -- 3.46)	**0.0031**	**0.0018**
Lack of staff for surgical care					
No	Ref.		Ref.		
Yes	1.35 (0.66 -- 2.76)	0.4118	1.25 (0.65 -- 2.41)	0.5103	0.8833
Within-subject correlation between delay type II and III	**−0.745**		***p*** **-value** **<** **.0001**		

Experience of delay type II was strongly and positively associated with having been referred due to complications, and with care-seeking at central hospitals compared to seeking care at a health center. The odds of delay type II among women who were referred were roughly two times that of women not referred.

A negative correlation was estimated between the probability of experiencing both delays type II and III. Delay type III is less likely to occur in a woman who experienced delay type II and vice-versa. A test on the overall effect of risk factors based on contrasts between model parameters showed that the significant effects on Table 3 were different between Delay II and Delay III.

## Discussion

This study evaluated the associations between two types of delays for a women experiencing pregnancy complications and the cause of death: delays type II, reaching a facility with capacity to manage pregnancy related complications and delays type III, delay in receiving appropriate treatment. This study focused on type II and III delays and therefore only provided the analysis of the deaths that occurred within health facilities.

The second delay was shown to be more frequent in this setting when compared with the third delay regardless of cause of death (direct or indirect). It was observed in more than 60% of women who died. The delay type II has been described in other studies [18–20]. Patient referral from a peripheral facility was strongly associated with the delay type II. This finding suggests that part of the delay in reaching the appropriate facility to treat the complication may occur at the peripheral health facility. At these facilities we often find junior and less qualified staff with critical limitations with regard to decision-making (diagnosis and treatment) in the face of an obstetric emergency. Another source of delays is related to the referral process management which is characterized by frequent fuel stock out and lack of ambulance maintenance or even inexistence of one functioning in the District. This slows down the decision-making to transfer patients and results in an inefficient functioning referral system. Referral has been noted elsewhere in Mozambique to be a risk factor for stillbirth outcomes [21]. A combination of factors such as the perception of poor quality of care and the lack of availability of skilled personnel have been identified in other studies to influence the choice of where to deliver and ultimately contributes to the delay for women to reach the health facility [22–25]. Elsewhere lack of transportation, prolonged travel time and seeking care at more than one facility were also identified as contributing factors to the delay type II [20].

Delay type II was identified and potentially contributed to one third of facility based maternal deaths. Women who died of acute direct obstetric complications were more likely to have faced delay type III. This might be interpreted as a lack of adequate emergency response from health services. Appropriate and timely complication management requires specific midwifery and specialist skills from the health providers as well as adequate availability of EmOC services [26–28]. Thus, availability of qualified services providers in right quantity must be combined with the availability of supplies for emergency care (e.g. drugs, blood), and the structural functioning of the health facilities such as the referral system and the functionality of the operating room [6, 27, 29–31].

We identified a negative relationship between delay II and delay III. A woman experiencing a delay in reaching the health facility may trigger a quick response from the health system when she arrives at the health facility and is less likely to experience a third delay. This correlation has been noted elsewhere [11]. This emphasizes the importance of ensuring the availability of services to identify and effectively manage the emergency resulting from pregnancy complications. Measures to improve delay type II tend to contribute to a reduction in delay III as well [32].

In a study in Brazil of maternal near miss cases the occurrence of any delay increased the severity of maternal outcome, particularly leading to maternal death [5]. Other authors have found an association between delays and severe pregnancy outcomes and maternal death [33].

In our study, direct obstetric causes were the most important causes of maternal deaths and hemorrhage the most frequent among this group followed by sepsis. HIV/AIDS was the most common cause of death among indirect causes. This finding aligns with the pattern of causes of maternal deaths in the developing world described elsewhere [5, 34].

In this national study 44% of the reviewed cases went directly from home to a health facility where they died. Another study conducted in a more urbanized setting in Mozambique found that 29% of near miss cases went directly from home to the health facility evaluated [14]. It is important to note that around 10% of the women who died were admitted to health facilities without any complication. Data from this group of women showed hemorrhage and obstructed labor as responsible of 42% of deaths. This results may indicate inadequate labor and delivery management including the issue of referral system discussed previously. Women still face important risk of dying even arriving in the maternities in healthier status [11]. The health facilities in this setting suffer from the same challenges described for the developing world in relation to human resources and the overall health services provision. A comprehensive effort to improve health system responsiveness is desirable for better maternal health outcomes.

### Study limitations

The absence of information on survival limited the analysis that might have further clarified factors that were determinants of maternal deaths. Also the fact that only part of the facility based data was available, limits the generalization of the conclusions to all country but rather to the covered facilities.

The quality of hospital and health center registers and medical charts was poor, as were storage and retrieval systems, which reduced our access to the files of all maternal deaths and to more complete information on the circumstance surrounding maternal deaths and the actions taken. The analysis done by the expert group helped reduce the inconsistency in the data from the files that were accessible.

This survey found that only 55% of the maternal deaths registered in the health facilities were reported in the national health information system. This study covered the analysis of around 32% of total institutional maternal deaths during the evaluation period.

## Conclusions

Investment is needed to strengthen referral linkages and secure hospital and health center readiness to rapidly diagnose and manage pregnancy related complications, and thus, lessen the impact of delays II and III, and ultimately reduce institutional maternal mortality. Further studies that include women who survive will be important to understand the broader impact of the delays on maternal health outcomes in this setting.

## Abbreviations

AIDS: Acquired immunodeficiency syndrome
BEmOC: Basic emergency obstetric care
CEmOC: Comprehensive emergency obstetric care
EmONC: Emergency obstetric neonatal care
HIV: Human immunodeficiency virus
MCH: Maternal and child health
ObGyn: Obstetric and gynecologist
SAS-STAT: Statistical analysis system
